# Risk of QTc prolongation with Chloroquine/Hyroxychloroquine and Azithromycin treatment for COVID‐19: Quantification and precautions for a busy clinician

**DOI:** 10.1002/joa3.12393

**Published:** 2020-07-03

**Authors:** Zeeshan Mansuri, Mahwish Adnan, Fatima Motiwala, Muhammad Khalid Zafar, Taranjeet Jolly, Shailesh Jain

**Affiliations:** ^1^ Department of Psychiatry Texas Tech University Health Science Center at Odessa/Permian Basin Odessa TX USA; ^2^ Central Michigan University Saginaw MI USA; ^3^ Department of Child and Adolescent Psychiatry Penn State College of Medicine Harrisburg PA USA

1

To the Editor,

Coronavirus (COVID‐19) has no proven effective treatment. However, several medications are being given off‐label to patients based on anecdotal reports and uncontrolled studies. Among these, the combination of Chloroquine (CHQ)/Hydroxychloroquine (H‐CHQ) and Azithromycin has garnered the maximum attention, owing partially to the support from the political leaders around the globe. This attention has led to significantly increased use of CHQ/H‐CHQ both inside and outside the hospital.^1^

With a busy clinician in mind, we will review and quantify the risk of QTc prolongation, arrhythmias, and torsades de pointes when using the combination of CHQ or H‐CHQ with Azithromycin. CHQ, H‐CHQ, and Azithromycin are known to produce QTc prolongation, torsade de pointes, and sudden cardiac death.^1^ H‐CHQ is associated with severe, sometimes fatal cardiomyopathy, atrio‐ventricular block, right or left bundle branch block, pulmonary hypertension, and sick sinus syndrome.^1^ This risk is exaggerated in older individuals with pre‐existing heart disease using other QTc prolonging drugs.^1^ The risk associated with the use of QTc prolonging medications for COVID‐19 may lead to a visible increase in unexpected cardiac death, particularly when they are utilized together or used with other medicines that also prolong the QTc interval.^1^

Rey et al in 2003, studied 46 rheumatology patients (42 using CHQ and 4 using H‐CHQ). 17.39% of patients developed a prolonged QTc interval. Such abnormalities were not linked to dose (*P* = .574), type of antimalarial drug (*P* = .452) and duration of drug use (*P* = .09).^2^ In the eight patients who's QTc was elevated, the average value was 0.456, which decreased to 0.410 on repeat echocardiogram after stopping CHQ for two weeks. None of the patients experienced an adverse cardiac event.^2^

A randomized placebo‐controlled parallel trial conducted in 2013 enrolled 116 healthy controls getting 1000 mg of chloroquine alone or in combination with escalating doses of Azithromycin (500, 1000, and 1500 mg daily). There was a definite correlation between azithromycin dose and QTc interval increase (Pearson *r* = 1, *P* = .01) in the dose range 500 mg (10 ms) to 1500 mg (14 ms).^3^

The Chinese expert Covid‐19 treatment consensus guidelines published on 20 February 2020, recommends several precautions with HCQ. These guidelines included baseline electrocardiography to rule out the QTc interval prolongation, avoidance of concomitant treatment of other QTC prolonging medications (ie, quinolones, macrolides, ondansetron) as well as various antiarrhythmic, antidepressant, and antipsychotic drugs.^4^

A parallel, randomized, double‐masked, phase IIb clinical trial from Brazil in April 2020 compared patients diagnosed with Covid‐19 being put on high dose CQ (600 mg CQ; 4 × 150 mg tablets twice daily for ten days; total dose 12 g) or low‐dosage CQ (450 mg CQ; 3 × 150 mg tablets and one placebo tablet twice daily on day 0, 3 × 150 mg tablets plus one placebo tablet once a day, then four placebo tablets from day 1 to day 4, then four placebo tablets twice daily from day 5 to day 9; total dose 2.7 g). 18.9% in the high dose group and 11.1% of the low dose group had prolonged QTc from normal to greater than 500 ms. There were no instances of ventricular tachycardia or torsade de pointes.^5^

The administration of H‐CHQ in the current setting of the COVID‐19 crisis is experimental. There are small trials that are already underway, but until we get more robust research, the use of H‐CHQ with or without Azithromycin is likely to be widespread. In the current review, we found that the risk of QTc prolongation when using CHQ/H‐CHQ alone or in combination with Azithromycin is between 10 and 40 ms for most cases in 11%–33% of the cases. The use of Azithromycin amplifies the risk of QTc prolongation. The critical increase from a normal QTc level to beyond 500 ms is rare in most studies unless there are additional risk factors. There were only sporadic instances of any arrhythmias or torsade de pointes, but since these patients had many underlying medical issues, a cause‐effect relationship cannot be established, and CHQ/H‐CHQ plus Azithromycin cannot be labeled as the main cause. In an ICU and in‐patient setting, intensive telemetry monitoring will help in possibly preventing torsades de pointes and sudden cardiac death. However, in an outpatient setting, we recommend that all clinicians, specifically psychiatrists and psychiatry practitioners, need to be aware of the risk of QTc prolongation associated with this medication so that they can advice their patients on the use of this medication in a better manner. The FDA has come out with a warning in April 2020, where it cautioned against the use of CHQ or H‐CHQ for Covid‐19 outside of the hospital setting or a clinical trial due to risk of heart rhythm problems.^1^ We recommend that all clinicals follow certain precautions to better help their patients:

1. Actively ask all patients about potential off label use of H‐CHQ and azithromycin or any other Covid‐19 medication that they potentially might have taken. This precaution is especially true for psychiatrists, and psychiatric practitioners as many psychotropic agents can potentially prolong the QTc interval.
2. Get regular EKG’s to monitor QTc's along with regular clinical monitoring and discuss emergency precautions in depth. Refer to a cardiologist as needed. Encourage the use of FDA approved mobile device like KardiaMobile to get the regular EKG’s if seeing the patient regularly is not possible.
3. Avoid other QTc prolonging medications to reduce the cumulative risk.^1^ Rule out electrolyte abnormalities, specifically hypokalemia, and hypomagnesemia, if clinically indicated.^1^
4. CHQ and H‐CHQ have long half‐lives ranging from 30 to 60 days, and so it can stay in the system for a while even after stopping it. Patients need to be aware of that.^1^
5. Educate all patients on the current data about CHQ/H‐CHQ and Azithromycin so that they are well informed about the risks and benefits associated with it.
6. CHQ and H‐CHQ remain pivotal drugs in the treatment of Malaria and other rheumatological disorders in many places in the world. The off label drug use of this drug can create significant drug shortages for patients who need these drugs for rheumatological reasons.^1^ Do not encourage off label use in mild cases of Covid‐19.
7. Warn about CHQ/H‐CHQ induced hemolytic anemia in patients with G6PD deficiency, the potential of causing hypoglycemia in diabetic patients, and of the higher risk of QTc prolongation in patients with renal insufficiency or failure.^1^

Off‐label use of CHQ, H‐CHQ, and Azithromycin has significantly increased in this pandemic in an attempt to reduce mortality and morbidity. The significant risk of QTc prolongation remains a serious concern. Clinicians need to take extra precautions in outpatient settings where cardiac monitoring might not be readily available.

REFERENCES1
Center for Drug Evaluation and Research, FDA
. FDA cautions use of hydroxychloroquine/chloroquine for COVID‐19. 2020 [cited 2020, April 26]. Available from https://www.fda.gov/drugs/drug‐safety‐and‐availability/fda‐cautions‐against‐use‐hydroxychloroquine‐or‐chloroquine‐covid‐19‐outside‐hospital‐setting‐or
2

Rey
LD
, 
Berneck
A
, 
Gonçalves
L
, 
Silva
MB
, 
Skare
TL
, 
Silva
JA
. Prolongamento do intervalo QT do eletrocardiograma em pacientes reumáticos usando antimaláricos. Rev Bras Reumatol Engl Ed. 2003;43(5):275–8.3
Pfizer Labs
. Zithromax (azithromycin tablets) and azithromycin for oral suspension. 2013 [cited 2020, March 28]. Available from http://www.accessdata.fda.gov/drugsatfda_docs/label/2013/050710s039,050711s036,050784s023lbl.pdf
4
Multicenter Collaboration Group of Department of Science and Technology of Guangdong Province and Health Commission of Guangdong Province for Chloroquine in the Treatment of Novel Coronavirus Pneumonia
. Expert consensus on chloroquine phosphate for the treatment of novel coronavirus pneumonia. Zhonghua Jie He He Hu Xi Za Zhi. 2020;43:E019–E29.3207536510.3760/cma.j.issn.1001-0939.2020.00195

Borba
MGS
, 
Val
FFA
, 
Sampaio
VS
, 
Alexandre
MAA
, 
Melo
GC
, 
Brito
M
, et al. Effect of high vs low doses of chloroquine diphosphate as adjunctive therapy for patients hospitalized with severe acute respiratory syndrome coronavirus 2 (SARS‐CoV‐2) infection: a randomized clinical trial. JAMA Netw Open. 2020;3(4):e208857.10.1001/jamanetworkopen.2020.8857PMC1212469132330277
